# The Role of Socioeconomic Status in the Association of Lung Function and Air Pollution—A Pooled Analysis of Three Adult ESCAPE Cohorts

**DOI:** 10.3390/ijerph16111901

**Published:** 2019-05-29

**Authors:** Dirk Keidel, Josep Maria Anto, Xavier Basagaña, Roberto Bono, Emilie Burte, Anne-Elie Carsin, Bertil Forsberg, Elaine Fuertes, Bruna Galobardes, Joachim Heinrich, Kees de Hoogh, Debbie Jarvis, Nino Künzli, Bénédicte Leynaert, Alessandro Marcon, Nicole Le Moual, Audrey de Nazelle, Christian Schindler, Valérie Siroux, Morgane Stempfelet, Jordi Sunyer, Sofia Temam, Ming-Yi Tsai, Raphaëlle Varraso, Bénédicte Jacquemin, Nicole Probst-Hensch

**Affiliations:** 1 Swiss Tropical and Public Health Institute, 4051 Basel, Switzerland; c.dehoogh@swisstph.ch (K.d.H.); nino.kuenzli@swisstph.ch (N.K.); christian.schindler@swisstph.ch (C.S.); nicole.probst@swisstph.ch (N.P.-H.); 2 Department of Public Health, University of Basel, 4001 Basel, Switzerland; 3 ISGlobal, 08003 Barcelona, Spain; josepm.anto@isglobal.org (J.M.A.); xavier.basagana@isglobal.org (X.B.); emilie.burte@inserm.fr (E.B.); anneelie.carsin@isglobal.org (A.-E.C.); jordi.sunyer@isglobal.org (J.S.); benedicte.jacquemin@isglobal.org (B.J.); 4 Hospital del Mar Medical Research Institute, 08003 Barcelona, Spain; 5 Department of Experimental and Health Sciences, Universitat Pompeu Fabra, 08002 Barcelona, Spain; 6 CIBER Epidemiología y Salud Pública, 08005 Barcelona, Spain; 7 Department of Public Health and Pediatrics, University of Turin, 10126 Turin, Italy; roberto.bono@unito.it; 8 INSERM, U1168, VIMA: Aging and Chronic Diseases, Epidemiological and Public Health Approaches, 94807 Villejuif, France; nicole.lemoual@inserm.fr (N.L.M.); STEMAM@mgen.fr (S.T.); raphaelle.varraso@inserm.fr (R.V.); 9 Univ Versailles St-Quentin-en-Yvelines, UMR-S 1168, 78180 Montigny le Bretonneux, France; 10 Public Health and Clinical Medicine, Umea University, University Hospital, 901 87 Umea, Sweden; bertil.forsberg@envmed.umu.se; 11 National Heart and Lung Institute, Imperial College London, London SW3 6LY, UK; e.fuertes@imperial.ac.uk (E.F.); d.jarvis@imperial.ac.uk (D.J.); 12 School of Social and Community Medicine, University of Bristol, Bristol BS8 1QU, UK; bruna.galobardes@gmail.com; 13 Institute of Epidemiology, German Research Center for Environmental Health (GmbH), Helmholtz Zentrum München, 85764 Neuherberg, Germany; heinrich@helmholtz-muenchen.de; 14 Institute and Outpatient Clinic for Occupational, Social and Environmental Medicine, Ludwig Maximilians University, 80336 Munich, Germany; 15 MRC-PHE Centre for Environment and Health, Imperial College London, London W2 1PG, UK; 16 INSERM UMR1152, Physiopathologie et épidémiologie des Maladies Respiratoires, équipe Epidémiologie, 75018 Paris, France; benedicte.leynaert@inserm.fr; 17 Unit of Epidemiology and Medical Statistics, Department of Diagnostics and Public Health, University of Verona, 37134 Verona, Italy; alessandro.marcon@univr.it; 18 Centre for Environmental Policy, Imperial College London, Exhibition Road, South Kensington Campus, London SW7 2AZ, UK; anazelle@imperial.ac.uk; 19 Institute for Advanced Biosciences, Inserm U1209, UMR CNRS 5309, Université Grenoble-Alpes, Team of Environmental Epidemiology Applied to Reproduction and Respiratory Health, 38700 Grenoble, France; valerie.siroux@ujf-grenoble.fr; 20 InVS, French Institute for Public Health Surveillance, 94415 Saint-Maurice, France; Morgane.STEMPFELET@santepubliquefrance.fr; 21 Department of Environmental and Occupational Health Sciences, University of Washington, Seattle, WA 98195, USA; mytsai2007@gmail.com

**Keywords:** Europe, socioeconomic position, air pollution, environmental equality, lung function

## Abstract

Ambient air pollution is a leading environmental risk factor and its broad spectrum of adverse health effects includes a decrease in lung function. Socioeconomic status (SES) is known to be associated with both air pollution exposure and respiratory function. This study assesses the role of SES either as confounder or effect modifier of the association between ambient air pollution and lung function. Cross-sectional data from three European multicenter adult cohorts were pooled to assess factors associated with lung function, including annual means of home outdoor NO_2_. Pre-bronchodilator lung function was measured according to the ATS-criteria. Multiple mixed linear models with random intercepts for study areas were used. Three different factors (education, occupation and neighborhood unemployment rate) were considered to represent SES. NO_2_ exposure was negatively associated with lung function. Occupation and neighborhood unemployment rates were not associated with lung function. However, the inclusion of the SES-variable education improved the models and the air pollution-lung function associations got slightly stronger. NO_2_ associations with lung function were not substantially modified by SES-variables. In this multicenter European study we could show that SES plays a role as a confounder in the association of ambient NO_2_ exposure with lung function.

## 1. Introduction

Ambient air pollution is the biggest contributor to the total environmental burden of the disease [1,2]. NO_2_ is estimated to be responsible for 68,000 premature deaths and 723,000 YLL (years of life lost) in the European Union in 2013 [3].

It has previously been shown that both air pollution and single SES parameters can have independent adverse effects on lung function and that SES can modify the effect of poor air quality on respiratory health [4,5,6,7,8]. However, SES is a multidimensional phenomenon which cannot be fully captured by a single socioeconomic variable (e.g., education, occupation, income) [9]. The assessment of the independent and joint association of SES and air pollution with health is complicated by the fact that there’s substantial variation in the relationship of air pollution exposure and individual SES in different cities and countries [4,10]. The role of socioeconomic and lifestyle factors as the confounder or effect modifier in the association between air pollution and health therefore remains a topic of great interest with regard to both causal inference and susceptibility related to air pollution health effects [11,12,13].

We investigated whether our previous ESCAPE (European Study of Cohorts for Air Pollution Effects) results on air pollution and lung function [14] were robust after the inclusion of further SES-variables. We then assessed whether SES influences the association between long-term exposure to traffic-related air pollution and respiratory function, using both individual-level (namely education and occupation) and neighborhood-level (unemployment rate) socioeconomic factors. This study uses pooled information from three adult respiratory cohorts which previously contributed data to the ESCAPE meta-analysis on air pollution and lung function [14] and the SESAP (Socioeconomic Status and Air Pollution in three European cohorts) pooled analysis on air pollution and SES [10]. In comparison to the first study [14], we additionally adjusted for the lifestyle variable pack-years of smoking and the SES-variables occupation and unemployment rate. The latter study [10] described the association between SES and NO_2_-exposure without considering health outcomes.

## 2. Materials and Methods 

### 2.1. Study Population

This is a cross-sectional study of 6502 adults who participated in the first follow-up of three Western European studies. Two of those studies share harmonized study protocols—the ECRHS (European Community Respiratory Health Survey) (n = 3772) [15] and the jointly initiated Swiss Study on Air Pollution and Lung and Heart Diseases (SAPALDIA) (n = 1922) [16]. Combined with EGEA (Epidemiological Study on the Genetics and Environment of Asthma) (n = 808) [17], these cohorts represent 22 study centers from eight European countries. Briefly, in ECRHS, more than 18,000 young adults aged 20 to 44 were recruited with an oversampling of asthmatics across Europe in 1991–1993 (ECRHS I) and 10,364 participated in the first follow-up (ECRHS II) between 2000 and 2002. SAPALDIA is a Swiss-wide cohort study covering three language regions. In 1991, 9651 participants aged 18 to 60 were recruited for a detailed interview and health examination (SAPALDIA 1). At the first follow-up (SAPALDIA 2), conducted in 2001–2003, 8047 participants provided health information. EGEA is a French case control and family-based study with 2047 participants including a group of asthmatic patients with their first-degree relatives and a group of control subjects recruited in the early 1990s (EGEA1: 1991–1995). At the first follow-up (EGEA2) conducted between 2003 and 2007, 1601 cohort subjects answered a detailed questionnaire. Many, but not all, of the centers in these three cohorts participated in the ESCAPE study [14]. In ESCAPE, harmonized air pollution measurement and modeling protocols were applied to estimate long-term exposure at the residential address of participants in the first study follow-up. The sample for the current analysis included participants who took part in the first follow-up of the three studies and who underwent lung function testing. The participants were from 20 urban areas in eight Western European countries, geographically spread across the North: Umea (Sweden), the central part: Ipswich, Norwich (United Kingdom); Erfurt (Germany); Antwerp (Belgium); Paris, Grenoble, Lyon (France); Geneva, Basel, Lugano (Switzerland) and the South of Europe: Marseille (France); Pavia, Turin, Verona (Italy); Albacete, Barcelona, Galdakao, Huelva, Oviedo (Spain). Paris and Grenoble hosted centers for both ECRHS and EGEA. Since participants of the same town but from different studies differed substantially according to air pollution exposure and SES variables the study areas were split into Paris (ECRHS), Paris (EGEA), Grenoble (ECRHS), and Grenoble (EGEA). This resulted in the final number of 22 study areas.

### 2.2. Air Pollution Exposure

**Figure 1:**
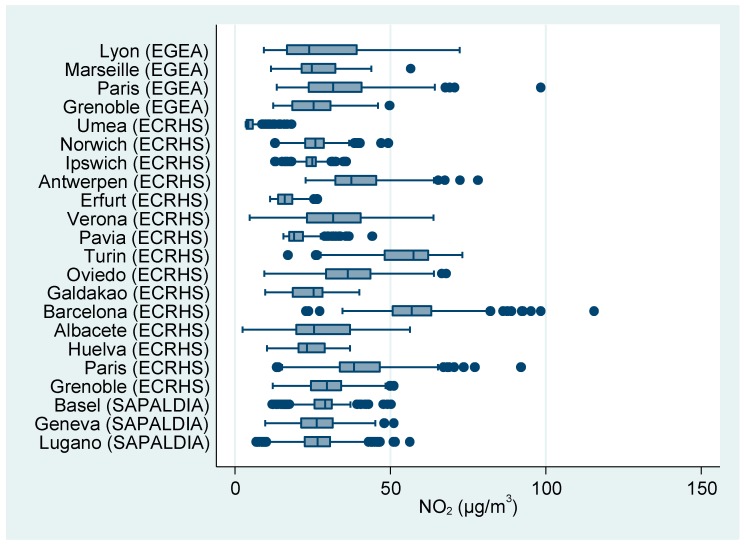
Boxplot of residential home outdoor nitrogen dioxide (NO_2_) annual mean concentrations at first follow-up by cohort and study center (all three cohorts).

The current study focused on modeled annual average nitrogen dioxide (NO_2_) at each participant’s place of residence as a marker of traffic-related air pollution since this variable was available in all study areas. ESCAPE methods to estimate the NO_2_ annual concentrations are described in detail elsewhere [18]. Briefly, between 2008 and 2011, two-week integrated NO_2_ passive sampler measurements at approximately 40 sites in each study area were conducted in three different seasons over a one-year period. Area-specific land use regression (LUR) models were developed to explain the spatial variation of NO_2_ using a variety of geographical data including traffic, population and land use variables. The LUR models were applied to estimate NO_2_ annual concentrations at each participant’s geocoded residential address at the first follow-up. The ESCAPE areas consist of small cities or metropolitan areas (larger cities with surrounding smaller suburban communities). The distribution of mean annual NO_2_ exposure by study area is presented in Figure 1.

### 2.3. Lung Function

Lung function was assessed by spirometry according to ATS recommendations [19]. All measuring instruments (ECRHS: Water-sealed bell spirometer (Biomedin, Padova, Italy) in most centers; SAPALDIA: Sensormedics 2200 SP (SensorMedics Corporation, Yorba Linda, CA, USA) in all centers; EGEA: Biomedin or SPIRODYN’R in most centers (see online supplement of [14] for details) were calibrated prior to each testing session. The technical personnel were specifically trained. In all three studies pre-bronchodilator measurements were performed. The lung function parameters considered in the present analyses are FVC (forced vital capacity) and FEV1 (forced expiratory volume in 1 s).

### 2.4. Socioeconomic Factors 

Individual SES was categorized according to education and occupation. Education was defined as a three-level categorical variable according to ESCAPE definitions: For EGEA and SAPALDIA, the questions on the highest attained degree were used (low: Primary or secondary school; medium: Middle or apprenticeship school; high: Technical college or university) whereas for ECRHS, the age of completion of full-time education was subclassified into low (≤16 years), medium (17–20 years) and high (≥21 years). Occupation was categorized into manual compared to non-manual work by classifying the corresponding ISCO-88 codes [20] of the longest held job between the baseline and follow-up. The ISCO-88 major groups six (Skilled agricultural and fishery workers), seven (Craft and related trades workers), eight (Plant and machine operators and assemblers), and nine (Elementary occupations) were defined as manual work. Neighborhood level unemployment rate was available in a reduced sample of n = 4766 subjects and n = 19 study areas and was grouped into area-specific tertiles to allow better comparability in the analyses across countries. The exact definition of this neighborhood level variable is described elsewhere [10].

### 2.5. Statistical Analysis

All analyses are based on the pooled data from the three cohorts. We applied multiple mixed linear models to analyze the role of SES in the associations between NO_2_ and lung function and used the study area as a random intercept. Regarding SES, we included the following variables: Educational level (three categories), occupation (manual compared to non-manual), and the neighborhood-level variable tertile of unemployment rate. Models were adjusted for the basic variables sex, age, age squared, height, height squared and additionally for the lifestyle variables smoking status (i.e., current, former, or never smoker), pack-years and pack-years squared (each with separate terms for current and former smokers), BMI (body mass index), and BMI squared. We systematically ran analyses for the full sample (n = 6502) and for a reduced sample (n = 4766) since the unemployment rate was not available for three study areas. Likelihood-ratio (LR) tests and AIC (Akaike Information Criterion) were used to assess the contribution of SES factors to the models. Effect modification of SES on the association of lung function and NO_2_ were tested by including interactions terms in the models. We carried out the following sensitivity analyses: a) A meta-analysis comparing and combining study area-specific results to assess heterogeneity of associations between study sites, b) a three-level model using neighborhood level further nested within the study area, c) a three-level model taking the family structure of EGEA into account, and d) interaction analyses excluding the lifestyle variables (smoking status, pack-years and BMI) to assess whether SES and NO_2_ interact when these possible mediator variables are excluded and thus might only be implicitly contained in SES. We used the statistical software Stata (StataCorp 2015. Stata Statistical Software: Release 14. StataCorp LP, College Station, TX, USA).

## 3. Results

Characteristics of the study population at the first follow-up are presented in Table 1. The average age of participants was 46 years, and was highest in SAPALDIA. EGEA and SAPALDIA had slightly more female (52.8% and 53.4%, respectively) than male subjects. Nearly half (47.6%) of the study participants were overweight or obese (i.e., had a BMI > 25 kg/m^2^). On average, 30% of the subjects reported current smoking. Compared to the other two cohorts, there were fewer subjects in the low education group in SAPALDIA whereas subjects in EGEA had the highest proportion of high education. Manual work was most common in ECRHS (21.7%). The median neighborhood level unemployment rate was 9.5%. For further analyses this variable was grouped into study area-specific tertiles. Mean FVC and FEV1 were 4284 mL and 3368 mL, respectively.

High-educated subjects were more exposed to NO_2_ and low-educated subjects had the lowest lung function. Subjects in the manual workforce had lower NO_2_ exposure and a slightly higher lung function than people in non-manual jobs. For tertile of unemployment rate there was a positive association with NO_2_ and a weak negative association with lung function (Table 2).

Associations between the SES-variables themselves are presented in Table 3. There is a strong relation between educational level and occupation, e.g., the proportion of subjects in the manual work force ranges from 4.3% for high education to 40.7% for low education. Subjects with low education or a manual occupation were the most likely to live in a neighborhood with an unemployment rate in the highest tertile.

Associations of NO_2_ with lung function from adjusted mixed linear models are presented in Table 4, both for the full and the reduced sample. Education was the only SES-variable that improved the model fit. We found rather strong evidence for associations between NO_2_ and the lung function in model M1 (basic and lifestyle variables), e.g., an adverse effect per 10 µg/m^3^ increment in NO_2_ in the full sample of −15.8 (−30.5; −1.2) mL for FVC and −11.3 (−23.8; 1.2) mL for FEV1. After including education in the models (Model M1 + education) the model fits improved and the estimates were stronger: −17.2 (−31.9; −2.6) mL for FVC and −12.7 (−25.2; −0.3) mL for FEV1. This corresponds with a 9% and 12% change in estimates for FVC and FEV1, respectively (e.g., by comparing the full sample estimates −15.8 mL and −17.2 mL for FVC) and is indicative of slight confounding by education. The models “Model M1 + education” have the lowest AIC for all combinations of the outcome and sample and are hence the best final models.

The parameter estimates for different education categories from the best models (i.e., Model M1 + education) are presented in Table 5. Subjects with medium or high education had significantly higher lung function values than the reference category of low education: 47.9 (7.3; 88.6) mL for medium education and 62.3 (20.6; 103.9) mL for high education for FVC and 53.6 (18.3; 88.9) mL for medium education and 71.5 (35.3; 107.7) mL for high education for FEV1. Therefore, education had an independent effect on lung function and it also improved the model fit according to the LR-test (FVC: *p* = 0.0128 and FEV1: *p* = 0.0005, respectively). The positive association between education and lung function was also confirmed in the reduced sample (results not shown).

We tested interaction terms of NO_2_ with education for both the full and the reduced sample. According to the AIC and the LR-Test the inclusion of these interactions did not improve the models, neither for FVC nor FEV1 (Table 6). Still, when looking at results stratified by education categories there was a tendency for stronger air pollution effects with increasing educational level (Table 7). As SES itself is unlikely to have a causal effect on lung function we also tested the interaction of NO_2_ with education in models excluding the lifestyle variables BMI, smoking status, and pack-years. These variables might act as mediators of the effect of SES on lung function. There was also no evidence of effect modification by SES in the models unadjusted for lifestyle (results not shown).

As our data are from 22 study centers in eight European countries, we tested the heterogeneity of the main results across centers. We performed a random−effect meta−analysis of study area−specific models for the full sample. As we found only little evidence of heterogeneity we performed a fixed−effects meta−analysis, which resulted in comparable effect estimates of −17.9 (−32.8; −2.9) mL change per 10 µg/m^3^ NO_2_ for FVC and −10.3 (−23.4; 2.9) for FEV1, with no evidence of heterogeneity for FEV1 (Higgins I^2^ = 0%) and only weak evidence of heterogeneity for FVC (I^2^ = 24.5%) (Supplement Figures S1 and S2). The three−level models with a) family (taking the family structure of EGEA into account) and b) neighborhood as a nested random factor within the study area showed very similar results as the main models (Supplement Table S1).

## 4. Discussion

In this pooled analysis of data from a subset of ESCAPE cohorts we confirmed that the previously reported inverse cross-sectional associations of NO_2_ with FVC and FEV1 [14] withstood more stringent adjustment for socioeconomic factors. The only SES-variable that improved the model fit was education. Education was independently and positively associated with lung function, and it slightly confounded the association of NO_2_ and lung function. We can only speculate about reasons why there was no evidence of an association of the other two SES-variables with lung function. It is possible that some manual professions might be beneficial to lung health (e.g., by being physically active or working outside), while other manual professions might expose workers to substances like dust and fumes and might therefore have negative effects. Unfortunately we did not have a variable for occupational exposure. As occupation is only a rough proxy of income, the effects of socio-economic factors on lung function may still have been underestimated. Unemployment rate at the community level might be influenced by factors going beyond the individual SES of the residents, e.g., by industrialization of the neighborhood [10].

From the perspective of causal inference and air quality regulation, it is essential to demonstrate the independence of adverse health effects of air pollution from potentially correlated health risk factors, including SES. The goal is to best possibly assess the causality of air pollution effects on health and well-being in order to justify investments into improved air quality. SES and poverty have been discussed for decades as important potential confounders or modifiers of air pollution-health associations [11,12,21,22]. This has stimulated our previous investigation into the association between air pollution and SES in different European cities [10], where we found the relationship of air pollution exposure and individual SES to strongly vary by city and country, as it had also been shown before [23]. Our results from geographically stratified analyses (Meta-Analyses, Supplement Table S1 and Figures S1 and S2) were quite homogeneous across study areas suggesting that these differences did not translate into differences in the air pollution-lung function association between study areas.

Our results add evidence towards a causal role of ambient air pollution in reducing lung function. It remains unclear whether NO_2_ health effects are clearly attributable to NO_2_ itself, or whether NO_2_ serves as a proxy for other polluting agents from combustion or traffic sources [24]. Whereas direct effects of NO_2_ on exacerbations of asthma have been established [25,26], the causal understanding of the independent effect of long-term exposure to NO_2_ on cardio-respiratory morbidity including lung function remains poor in the light of the small number of studies investigating two- or even three-pollutant models [27].

From the perspective of health promotion it is not sufficient to consider air pollution effects in isolation [28]. The identification of factors that modify the susceptibility to air pollution is important. For example, we have previously shown that the respiratory function of obese persons may be more susceptible to inhaled pollutants from traffic exhaust [14] and may be less likely to recover after air quality improves [29]. These results point to the possibility that the benefits of improved air quality on respiratory health may be diminished by an uncontrolled obesity epidemic. Several studies previously demonstrated stronger adverse effects of air pollution on health in subjects with lower SES [11,12,21,22,28]. Brunt et al. [28] reported stronger NO_2_, PM10 and PM2.5 effects on respiratory disease mortality for the more deprived small geographic areas of Wales, whereas no associations were observed in the least deprived areas. Deprivation status of the geographic areas was itself strongly associated with respiratory disease mortality. This was in accordance with the general evidence for an almost 10 year difference in life-expectancy between the most and least deprived areas in the UK, which has so far been attributed largely to behaviors [30]. The results by [28] suggest that differences in environmental conditions may contribute to health differences by deprivation state. In the current study focusing on lung function as an endpoint, we did not observe statistically significant interactions between air pollution and the SES-variable education. If anything, there was a tendency for stronger air pollution effects in more educated persons irrespective of adjustment for lifestyle factors. But more educated persons still had better lung function. It remains unclear if this could be due to other lifestyle variables like physical activity and diet, which were not available in the current analysis. Future studies need to further clarify the complex interplay between SES, physical activity [31], additional lifestyle factors, and air pollution on lung function.

We observed independent associations of education with lung function. Those were larger than the effects of NO_2_ across the contrast observed in air quality. These SES-lung function associations may be partly explained by a correlation of low SES with occupational settings that promote respiratory diseases [32] and that are not well captured by an occupational variable merely differentiating between manual and non-manual work. In the current study the latter variable was not associated with lung function. The education-lung function association could also be mediated by behaviors that are themselves bad for lung health (e.g., poor nutrition, physical inactivity, obesity, smoking) [6,12]. As the positive association of SES with lung function was limited to education, this individual SES parameter seems to best capture mediators of lung function in our study.

Our study has several advantages. It covers a wide range of urban areas representing different regions of Western Europe. NO_2_ exposure was estimated with the same methodology for all centers, which makes our results comparable across studies and centers. The centers included cover a wide range of NO_2_ exposure, with estimated annual means ranging from 5.3 µg/m^3^ (Umea, Sweden) to 58.2 µg/m^3^ (Barcelona, Spain) (Figure 1). The protocols and study designs of the three studies were quite similar. However, the current study is cross-sectional by design and we can therefore not differentiate whether people with low lung function and respiratory problems are less likely to succeed professionally and thus live in more deprived areas or whether low educational achievements and jobs and poor residential conditions lead to poor lung function later in life. There may be several explanations for why we did not observe statistically significant effect modification of the NO_2_-lung function associations by the SES variable education. It may reflect limitations in statistical power or in the data available to classify participants’ SES. For example, we only had educational level and occupation available for individual SES classification. This null result may also reflect the true absence of effect modification.

## 5. Conclusions

These results provide further evidence that the inverse association between traffic-related air pollution and lung function is not the result of confounding by SES. Consequently, it is likely that improvements in air quality would benefit lung health [33,34] in the entire population and might even help to reduce inequalities in health. Health benefits of improved air quality have been shown by many studies, e.g., for Lausanne, Switzerland, with a reduction of 1%–2% in all-cause annual mortality attributable to a decrease in NO_2_ [35]. A health impact analysis for Barcelona estimated a beneficial effect of 3500 fewer deaths as a result of reducing the mean PM10 exposure (50 µg/m^3^) to the annual mean value recommended by the WHO (20 µg/m^3^) [36]. Beneficial effects of different scenarios for PM2.5 reduction were also shown for 26 European cities [37]. The large potential impact of policy efforts for clean air can also be seen in the US, where the success in reducing air pollution was estimated to have prevented 160,000 cases of premature mortality and 130,000 heart attacks between 1990 and 2010 [38]. This is strong support for the call to develop global evidence-based clean air standards [39]. Given the importance of the topic from a health-in-all-policy perspective, factors determining susceptibility to air pollution must be studied further.

## Figures and Tables

**Table 1:** Characteristics of the study population at the first follow-up.

		EGEA	ECRHS	SAPALDIA	Total
Year		2003–2007	2000–2002	2001–2003	
N		808	3772	1922	6502
Age		44.9 ± 16	42.6 ± 7.2	52.7 ± 11.4	45.9 ± 11
Height (cm)		168.4 ± 8.7	169 ± 9.4	168.7 ± 9.2	168.8 ± 9.3
NO_2_ (µg/m^3^)		29 ± 12.3	29.6 ± 15.7	26.9 ± 6.8	28.7 ± 13.3
FVC (mL)		4172 ± 1057	4385 ± 994	4130 ± 1028	4284 ± 1019
FEV1 (mL)		3245 ± 908	3511 ± 803	3136 ± 830	3368 ± 842
Pack–years		0 (0; 6.6)	3.5 (0; 18)	2 (0; 20)	2.1 (0; 17.3)
Sex	Women	52.8%	48.7%	53.4%	50.6%
BMI	Underweight (<18.5)	3.1%	1.6%	2.2%	2.0%
	Normal (18.5–25)	58.4%	49.4%	49.0%	50.4%
	Overweight (25–30)	28.6%	35.6%	34.7%	34.4%
	Obese(>30)	9.9%	13.5%	14.2%	13.2%
Smoking	Current	21.5%	32.2%	28.0%	29.6%
	Former	28.3%	27.1%	31.9%	28.7%
	Never	50.1%	40.6%	40.1%	41.7%
Education	Low	25.5%	26.6%	6.1%	20.4%
	Medium	23.6%	35.6%	63.7%	42.4%
	High	50.9%	37.8%	30.2%	37.2%
Occupation	manual	9.7%	21.7%	16.5%	18.7%
Neighborhood unemployment rate (%) ^1^		8.8 (6.5; 11.3)	10.9 (6.8; 16)	4.5 (3.4; 5.3)	9.5 (5.8; 14.4)

For continuous variables mean ± standard deviation are presented, except for pack-years and neighborhood unemployment rate where the median (first quartile; third quartile) is presented. ^1^ Unemployment rate on neighborhood level is only available in the reduced sample of n = 4766.

**Table 2:** Unadjusted relations of socioeconomic status (SES)-variables with NO_2_ and lung function.

		NO_2_ (µg/m^3^)	FVC (mL)	FEV1 (mL)
Education	Low	28.4 ± 13.5	4078 ± 994	3174 ± 816
	Medium	28.0 ± 12.3	4213 ± 1016	3300 ± 842
	High	29.8 ± 14.1	4478 ± 1003	3552 ± 821
Occupation	non-manual	29.2 ± 13.3	4264 ± 999	3359 ± 829
	Manual	26.5 ± 12.9	4371 ± 1099	3409 ± 895
Neighborhood	Low	27.1 ± 14.3	4393 ± 1034	3461 ± 846
unemployment	Medium	30.4 ± 14.2	4323 ± 1024	3421 ± 837
rate tertile	High	31.8 ± 15.0	4294 ± 984	3403 ± 815

Presented are mean ± standard deviation. n = 6502 (education and occupation) and n = 4766 (unemployment rate). Unemployment rate was categorized into area-specific tertiles.

**Table 3:** Cross-tabulation of SES-variables.

			Tertiles of Unemployment Rate			Occupation	
			Low	Medium	High	Non-Manual	Manual
**Educational level**	**low**	N	355	407	481	737	506
		%	28.6%	32.7%	38.7%	59.3%	40.7%
	**medium**	N	539	520	531	1283	307
		%	33.9%	32.7%	33.4%	80.7%	19.3%
	**high**	N	715	666	552	1850	83
		%	37.0%	34.5%	28.6%	95.7%	4.3%
**Occupation**	**non-manual**	N	1365	1304	1201		
		%	35.3%	33.7%	31.0%		
	**manual**	N	244	289	363		
		%	27.2%	32.3%	40.5%		

Presented are n’s and row percentages in the reduced sample (n = 4766). Pearson’s Chi-squared-Test for all three cross-tables: *p* < 0.001.

**Table 4:** Associations of lung function with NO_2_, results of adjusted mixed effect models.

Outcome	Sample	Model	NO_2_	(95% CI)	*p*−Value	AIC
FVC	Full	Basic and lifestyle variables (Model M1)	−15.8	(−30.5; −1.2)	0.034	100,256.3
FVC	Full	Model M1 + education	−17.2	(−31.9; −2.6)	0.021	100,251.6
FVC	Full	Model M1 + occupation	−15.9	(−30.6; −1.3)	0.033	100,258.3
FVC	Full	Model M1 + all SES−variables	−17.1	(−31.8; −2.4)	0.022	100,253.2
FVC	Reduced	Basic and lifestyle variables (Model M1)	−18.6	(−34.3; −2.9)	0.02	73,571.5
FVC	Reduced	Model M1 + education	−20.3	(−36; −4.6)	0.011	73,568.4
FVC	Reduced	Model M1 + occupation	−18.6	(−34.3; −2.9)	0.021	73,573.5
FVC	Reduced	Model M1 + education + occupation	−20.0	(−35.7; −4.2)	0.013	73,569.4
FVC	Reduced	Model M1 + unemployment tertile	−16.6	(−32.6; −0.7)	0.041	73,573.9
FVC	Reduced	Model M1 + all SES−variables	−18.2	(−34.3; −2.2)	0.026	73,572.2
FEV1	Full	Basic and lifestyle variables (Model M1)	−11.3	(−23.8; 1.2)	0.077	98,381.6
FEV1	Full	Model M1 + education	−12.7	(−25.2; −0.3)	0.046	98,370.5
FEV1	Full	Model M1 + occupation	−11.7	(−24.2; 0.8)	0.067	98,381.5
FEV1	Full	Model M1 + all SES−variables	−12.8	(−25.3; −0.3)	0.045	98,372.4
FEV1	Reduced	Basic and lifestyle variables (Model M1)	−14.4	(−28; −0.8)	0.038	72,427.9
FEV1	Reduced	Model M1 + education	−16.4	(−30; −2.8)	0.018	72,418.1
FEV1	Reduced	Model M1 + occupation	−15.1	(−28.7; −1.5)	0.03	72,427.6
FEV1	Reduced	Model M1 + education + occupation	−16.5	(−30.1; −2.9)	0.017	72,420.0
FEV1	Reduced	Model M1 + unemployment tertile	−13.6	(−27.4; 0.3)	0.054	72,431.5
FEV1	Reduced	Model M1 + all SES−variables	−16.2	(−30; −2.3)	0.023	72,423.9

n = 6502 (full sample), n = 4766 (reduced sample with available unemployment rate). Each line shows results from one model. Results are presented in mL per 10 µg/m^3^ NO_2_. Basic variables include sex, age, age squared, height and height squared. Lifestyle variables are smoking, pack-years, pack-years squared, interaction of smoking and pack-years, interaction of smoking and pack-years squared, body mass index (BMI), and BMI squared. Study center was included as a random intercept. A decrease in (akaike information criterion) AIC can be interpreted as an improvement of the model fit.

**Table 5:** Independent association of education with lung function.

Outcome	SES	Group	Estimate (mL)	(95% CI)	*p*-Value
FVC	Education	Low	-	reference	
FVC		Medium	47.9	(7.3; 88.6)	0.021
FVC		High	62.3	(20.6; 103.9)	0.003
FEV1	Education	Low	-	reference	
FEV1		Medium	53.6	(18.3; 88.9)	0.003
FEV1		High	71.5	(35.3; 107.7)	0.000

n = 6502 (full sample). Presented are effect estimates for education from the models “Model M1 + education” from Table 4. Included in the models are the basic variables sex, age, age squared, height and height squared and additionally the lifestyle variables smoking, pack-years, pack-years squared, interaction of smoking and pack-years, interaction of smoking and pack-years squared, BMI, and BMI squared, and NO_2_. Study center was included as a random intercept.

**Table 6:** Assessment of interaction between NO_2_ and education on lung function.

	Outcome	Sample	AIC	*p*-Value
Model M1 + education	FVC	Full	100,251.6	
Model M1 + education + NO_2_*education	FVC	Full	100,254.2	0.49
Model M1 + education	FVC	Reduced	73,568.4	
Model M1 + education + NO_2_*education	FVC	Reduced	73,569.8	0.27
Model M1 + education	FEV1	Full	98,370.5	
Model M1 + education + NO_2_*education	FEV1	Full	98,372.3	0.34
Model M1 + education	FEV1	Reduced	72,418.1	
Model M1 + education + NO_2_*education	FEV1	Reduced	72,418.8	0.20

For each of the two outcomes (forced vital capacity) FVC and (forced expiratory volume in 1 s) FEV1 the interaction of NO_2_ and education was tested in both the full and the reduced sample. The first line lists AIC of the best model (M1 + education as presented in Table 4) and the second line gives the AIC and the p-value for the final model including the interaction. The p-values are from likelihood-ration (LR)-Tests testing the interaction terms. A decrease in AIC can be interpreted as an improvement of the model fit.

**Table 7:** Adjusted association of NO_2_ with lung function, stratified by education.

Outcome	Sample	SES	Group	Estimate	(95% CI)	*p*−Value
FVC	full	education	low	−5.6	(−29.9; 18.7)	0.653
FVC			medium	−19.5	(−39.8; 0.7)	0.059
FVC			high	−22.0	(−41; −2.9)	0.024
FVC	reduced	education	low	−8.3	(−33; 16.4)	0.510
FVC			medium	−16.8	(−39.1; 5.5)	0.139
FVC			high	−30.2	(−50.4; −10.1)	0.003
FEV1	full	education	low	−0.5	(−21.5; 20.5)	0.962
FEV1			medium	−14.5	(−31.9; 3)	0.104
FEV1			high	−18.3	(−34.7; −1.9)	0.028
FEV1	reduced	education	low	−1.8	(−23.5; 19.8)	0.868
FEV1			medium	−17.5	(−37; 1.9)	0.077
FEV1			high	−24.7	(−42.3; −7.1)	0.006

n = 6502 (full sample). Results are presented in mL per 10 µg/m^3^ NO_2_. Included in the models are the basic variables sex, age, age squared, height and height squared and additionally the lifestyle variables smoking, pack-years, pack-years squared, interaction of smoking and pack-years, interaction of smoking and pack-years squared, BMI, and BMI squared, and NO_2_. Study center was included as a random intercept.

## Supplementary Materials

The following are available online at https://www.mdpi.com/1660-4601/16/11/1901/s1; Figure S1: Meta-analysis of the association of NO_2_ with FVC in the full sample, by study center and overall; Figure S2: Meta-analysis of the association of NO_2_ with FEV1 in the full sample, by study center and overall; Table S1: Sensitivity analyses.

## References

[1] WHO (2016). Ambient Air Pollution: A Global Assessment of Exposure and Burden of Disease.

[2] Cohen A.J., Brauer M., Burnett R., Anderson H.R., Frostad J., Estep K., Balakrishnan K., Brunekreef B., Dandona L., Dandona R. (2017). Estimates and 25-year trends of the global burden of disease attributable to ambient air pollution: An analysis of data from the Global Burden of Diseases Study 2015. Lancet.

[3] EEA (2016). Air Quality in Europe—2016 Report. European Environment Agency Report No 28/2016.

[4] Wheeler B.W., Ben-Shlomo Y. (2005). Environmental equity, air quality, socioeconomic status, and respiratory health: A linkage analysis of routine data from the Health Survey for England. J. Epidemiol. Community Health.

[5] O’Neill M.S., Jerrett M., Kawachi I., Levy J.I., Cohen A.J., Gouveia N., Wilkinson P., Fletcher T., Cifuentes L., Schwartz J. (2003). Health, wealth, and air pollution: Advancing theory and methods. Environ. Health Perspect..

[6] Hegewald M.J., Crapo R.O. (2007). Socioeconomic status and lung function. Chest.

[7] McFadden E., Luben R., Wareham N., Bingham S., Khaw K.T. (2009). How far can we explain the social class differential in respiratory function? A cross-sectional population study of 21,991 men and women from EPIC-Norfolk. Eur. J. Epidemiol..

[8] Schikowski T., Sugiri D., Reimann V., Pesch B., Ranft U., Krämer U. (2008). Contribution of smoking and air pollution exposure in urban areas to social differences in respiratory health. BMC Public Health.

[9] Braveman P.A., Cubbin C., Egerter S., Chideya S., Marchi K.S., Metzler M., Posner S. (2005). Socioeconomic status in health research: One size does not fit all. JAMA.

[10] Temam S., Burte E., Adam M., Antó J.M., Basagaña X., Bousquet J., Carsin A.E., Galobardes B., Keidel D., Künzli N. (2017). Socioeconomic position and outdoor nitrogen dioxide (NO_2_) exposure in Western Europe: A multi-city analysis. Environ. Int..

[11] Chi G.C., Hajat A., Bird C.E., Cullen M.R., Griffin B.A., Miller K.A., Shih R.A., Stefanick M.L., Vedal S., Whitsel E.A. (2016). Individual and Neighborhood Socioeconomic Status and the Association between Air Pollution and Cardiovascular Disease. Environ Health Perspect..

[12] Laurent O., Bard D., Filleul L., Segala C. (2007). Effect of socioeconomic status on the relationship between atmospheric pollution and mortality. J. Epidemiol. Community Health.

[13] Humphrey J.L., Lindstrom M., Barton K.E., Shrestha P.M., Carlton E.J., Adgate J.L., Miller S.L., Root E.D. (2019). Social and Environmental Neighborhood Typologies and Lung Function in a Low-Income, Urban. Population. Int. J. Environ. Res. Public Health.

[14] Adam M., Schikowski T., Carsin A.E., Cai Y., Jacquemin B., Sanchez M., Vierkötter A., Marcon A., Keidel D., Sugiri D. (2015). Adult lung function and long-term air pollution exposure. ESCAPE: A multicentre cohort study and meta-analysis. Eur. Respir. J..

[15] Jarvis D. (2002). The European Community Respiratory Health Survey II. Eur. Respir. J..

[16] Ackermann-Liebrich U., Kuna-Dibbert B., Probst-Hensch N.M., Schindler C., Dietrich D.F., Stutz E.Z., Bayer-Oglesby L., Baum F., Brändli O., Brutsche M. (2005). Follow-up of the Swiss Cohort Study on Air Pollution and Lung Diseases in Adults (SAPALDIA 2) 1991–2003: Methods and characterization of participants. Soz Praventivmed..

[17] Siroux V., Boudier A., Bousquet J., Bresson J.L., Cracowski J.L., Ferran J., Gormand F., Just J., Le Moual N., Morange S. (2009). Phenotypic determinants of uncontrolled asthma. J. Allergy Clin. Immunol..

[18] Beelen R., Hoek G., Vienneau D., Eeftens M., Dimakopoulou K., Pedeli X., Tsai M.Y., Künzli N., Schikowski T., Marcon A. (2013). Development of NO_2_ and NOx land use regression models for estimating air pollution exposure in 36 study areas in Europe—The ESCAPE project. Atmos. Environ..

[19] ATC (1995). Standardization of Spirometry, 1994 Update. American Thoracic Society. Am. J. Respir. Crit. Care Med..

[20] ISCO88 (1991). International Standard Classification of Occupations.

[21] Deguen S., Zmirou-Navier D. (2010). Social inequalities resulting from health risks related to ambient air quality—A European review. Eur. J. Public Health.

[22] Forastiere F., Stafoggia M., Tasco C., Picciotto S., Agabiti N., Cesaroni G., Perucci C.A. (2007). Socioeconomic status, particulate air pollution, and daily mortality: Differential exposure or differential susceptibility. Am. J. Ind. Med..

[23] Padilla C.M., Kihal-Talantikite W., Vieira V.M., Rossello P., Le Nir G., Zmirou-Navier D., Deguen S. (2014). Air quality and social deprivation in four French metropolitan areas—A localized spatio-temporal environmental inequality analysis. Environ. Res..

[24] Kutlar Joss M., Dyntar D., Rapp R. (2015). Gesundheitliche Wirkungen der NO_2_-Belastung auf den Menschen: Synthese der Neueren Literatur auf Grundlage des WHO-REVIHAAP Berichts.

[25] EPA (2016). Integrated Science Assessment for Oxides of Nitrogen—Health Criteria.

[26] Muñoz X., Barreiro E., Bustamante V., Lopez-Campos J.L., González-Barcala F.J., Cruz M.J. (2019). Diesel exhausts particles: Their role in increasing the incidence of asthma. Reviewing the evidence of a causal link. Sci. Total Environ..

[27] WHO (2013). Health Risks of Air Pollution in Europe—HRAPIE Project.

[28] Brunt H., Barnes J., Jones S.J., Longhurst J.W.S., Scally G., Hayes E. (2016). Air pollution, deprivation and health: Understanding relationships to add value to local air quality management policy and practice in Wales, UK. J. Public Health (Oxf.).

[29] Schikowski T., Schaffner E., Meier F., Phuleria H.C., Vierkötter A., Schindler C., Kriemler S., Zemp E., Krämer U., Bridevaux P.O. (2013). Improved air quality and attenuated lung function decline: Modification by obesity in the SAPALDIA cohort. Environ. Health Perspect..

[30] Marmot M., Bell R. (2012). Fair society, healthy lives. Public Health.

[31] Fuertes E., Markevych I., Jarvis D., Vienneau D., de Hoogh K., Antó J.M., Bowatte G., Bono R., Corsico A.G., Emtner M. (2018). Residential air pollution does not modify the positive association between physical activity and lung function in current smokers in the ECRHS study. Environ. Int..

[32] ILO (2010). ILO List of Occupational Diseases.

[33] Downs S.H., Schindler C., Liu L.J.S., Keidel D., Bayer-Oglesby L., Brutsche M.H., Gerbase M.W., Keller R., Künzli N., Leuenberger P. (2007). Reduced exposure to PM10 and attenuated age-related decline in lung function. N. Engl. J. Med..

[34] Schindler C., Keidel D., Gerbase M.W., Zemp E., Bettschart R., Brandli O., Brutsche M.H., Burdet L., Karrer W., Knopfli B. (2009). Improvements in PM10 exposure and reduced rates of respiratory symptoms in a cohort of Swiss adults (SAPALDIA). Am. J. Respir. Crit. Care Med..

[35] Castro A., Kunzli N., Gotschi T. (2017). Health benefits of a reduction of PM10 and NO_2_ exposure after implementing a clean air plan in the Agglomeration Lausanne-Morges. Int. J. Hyg. Environ. Health.

[36] Perez L., Sunyer J., Kunzli N. (2009). Estimating the health and economic benefits associated with reducing air pollution in the Barcelona metropolitan area (Spain). Gac. Sanit..

[37] Ballester F., Medina S., Boldo E., Goodman P., Neuberger M., Iñiguez C., Künzli N. (2008). Reducing ambient levels of fine particulates could substantially improve health: A mortality impact assessment for 26 European cities. J. Epidemiol. Community Health.

[38] EPA (2011). The Benefits and Costs of the Clean Air Act from 1990 to 2020.

[39] Kutlar Joss M., Eeftens M., Gintowt E., Kappeler R., Künzli N. (2017). Time to harmonize national ambient air quality standards. Int. J. Public Health.

