# Supplementary material for: The Role of Socioeconomic Status in the Association of Lung Function and Air Pollution—A Pooled Analysis of Three Adult ESCAPE Cohorts
Source: Int J Environ Res Public Health. 2019 May 29;16(11):1901. doi: 10.3390/ijerph16111901 (PMC6603717; doi:10.3390/ijerph16111901)
Supplement: Supplementary file 1 [file ijerph-16-01901-s001.pdf]

# Supplementary Materials: The role of socioeconomic status in the association of lung function and air pollution– a pooled analysis of three adult ESCAPE cohorts

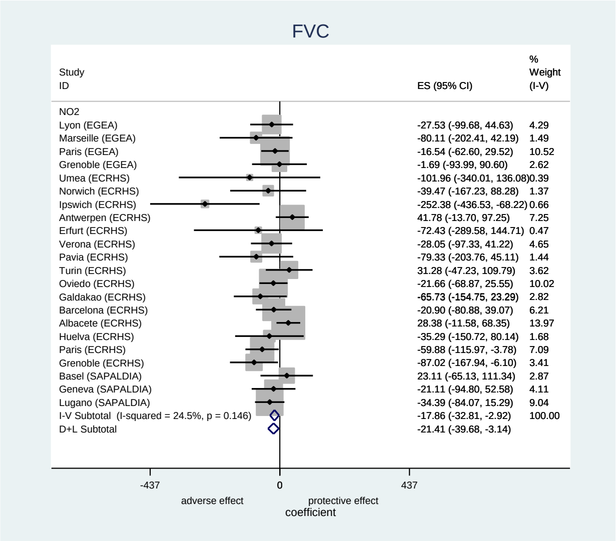


**Figure S1.** Meta-analysis of the association of $NO_2$ with FVC in the full sample, by study center and overall Forest plot displaying the study area-specific mixed linear regression model estimates of the association of $NO_2$ (per 10 μg/m$^3$ increment) with FVC. I-squared: Variation in estimated effects attributable to heterogeneity. I-V Subtotal: Fixed effects model using the inverse variance method, D+L Subtotal: Random effects model using the DerSimonian and Laird method. The mixed linear regression models were adjusted for age, age squared, height, height squared, sex, BMI, BMI squared, smoking status, pack-years, interaction of smoking status and pack-years, pack-years squared, interaction of smoking status and pack-years squared, and education (models M1 + education). Negative estimates indicate lower lung function with increasing exposure. ES: Effect size.

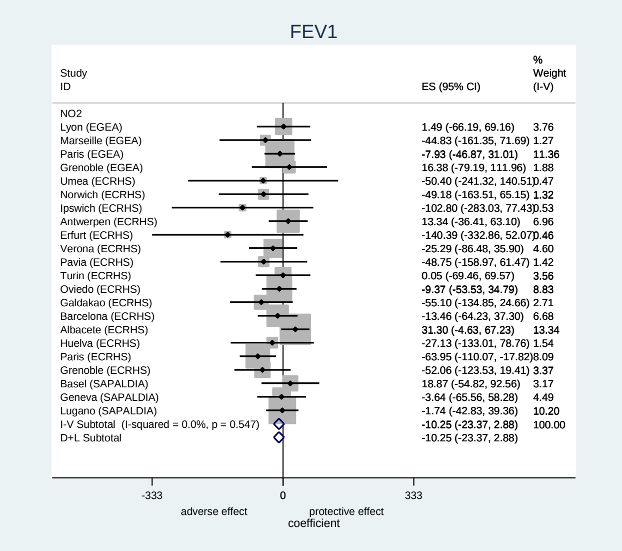


**Figure S2.** Meta-analysis of the association of $NO_2$ with FEV1 in the full sample, by study center and overall. Forest plot displaying the study area-specific mixed linear regression model estimates of the association of $NO_2$ (per 10 μg/m$^3$ increment) with FEV1. I-square: Variation in estimated effects

attributable to heterogeneity. I-V Subtotal: Fixed effects model using the inverse variance method, D+L Subtotal: Random effects model using the DerSimonian and Laird method. The mixed linear regression models were adjusted for age, age squared, height, height squared, sex, BMI, BMI squared, smoking status, pack-years, interaction of smoking status and pack-years, pack-years squared, interaction of smoking status and pack-years squared, and education (models M1 + education). Negative estimates indicate lower lung function with increasing exposure. ES: Effect size.

**Table S1.** Sensitivity analyses.

| Outcome | Sample | Model | $NO_2$ | (95% CI) | p−value |
|---|---|---|---|---|---|
| FVC | Full | Meta−Analysis (fixed) | −17.9 | (−32.8; −2.9) | 0.019 |
| FVC | Full | Meta−Analysis (random) | −21.4 | (−39.7; −3.1) | |
| FVC | Full | All variables (three−level pooled model)[1] | −16.3 | (−31.2; −1.5) | 0.031 |
| FVC | Reduced | All variables (three−level pooled model)[2] | −19.2 | (−34.9; −3.5) | 0.017 |
| FEV1 | Full | Meta−Analysis (fixed) | −10.3 | (−23.4; 2.9) | 0.126 |
| FEV1 | Full | Meta−Analysis (random) | −10.3 | (−23.4; 2.9) | |
| FEV1 | Full | All variables (three−level pooled model)[1] | −12.3 | (−24.9; 0.2) | 0.054 |
| FEV1 | Reduced | All variables (three−level pooled model)[2] | −16.3 | (−30.2; −2.4) | 0.022 |

n = 6502 (full sample), n = 4766 (reduced sample). For the three-level-models [1] family and [2] neighborhood respectively were considered as additional random effect nested in study area.

© 2019 by the authors; licensee MDPI, Basel, Switzerland. This article is an open access article distributed under the terms and conditions of the Creative Commons by Attribution (CC-BY) license (http://creativecommons.org/licenses/by/4.0/).
